# Locally-advanced primary neuroendocrine carcinoma of the breast: case report and review of the literature

**DOI:** 10.1186/1477-7819-11-128

**Published:** 2013-06-05

**Authors:** Fernando A Angarita, Jorge L Rodríguez, Eugenio Meek, Jesus O Sánchez, Mauricio Tawil, Lilian Torregrosa

**Affiliations:** 1 Department of Surgery, Hospital Universitario San Ignacio, Pontificia Universidad Javeriana, Carrera 7 Nº 40 - 62, Oficina 718, Bogotá, Colombia; 2 Department of Pathology, Hospital Universitario San Ignacio, Pontificia Universidad Javeriana, Bogotá, Colombia; 3 Department of Oncology, Centro Javeriano de Oncología, Bogotá, Colombia; 4 Breast and Soft Tissue Clinic, Centro Javeriano de Oncología, Bogotá, Colombia; 5 Division of Experimental Therapeutics, Toronto General Research Institute, University Health Network, Toronto, ON, Canada; 6 Institute of Medical Science, University of Toronto, Toronto, ON, Canada

**Keywords:** Breast neoplasm, Neuroendocrine tumor, Chromogranin A, Synaptophysin

## Abstract

**Background:**

Primary neuroendocrine carcinoma of the breast is a heterogeneous group of rare tumors with positive immunoreactivity to neuroendocrine markers in at least 50% of cells. Diagnosis also requires that other primary sites be ruled out and that the same tumor show histological evidence of a breast *in situ* component. Primary neuroendocrine carcinoma of the breast rarely presents as locally advanced disease and less frequently with such widespread metastatic disease as described herein. The review accompanying this case report is the first to provide an overview of all the cases of primary neuroendocrine carcinoma of the breast published in the literature and encompasses detailed information regarding epidemiology, histogenesis, clinical and histologic diagnosis criteria, classification, surgical and adjuvant treatment, as well as prognosis. We also provide recommendations for common clinical and histologic pitfalls associated with this tumor.

**Case presentation:**

We describe a case of a 51-year-old Hispanic woman initially diagnosed with locally-advanced invasive ductal carcinoma that did not respond to neodjuvant treatment. After undergoing modified radical mastectomy the final surgical pathology showed evidence of alveolar-type primary neuroendocrine carcinoma of the breast. The patient was treated with cisplatin/etoposide followed by paclitaxel/carboplatinum. Thirteen months after surgery the patient is alive, but developed pulmonary, bone, and hepatic metastasis.

**Conclusion:**

The breast *in situ* component of primary neuroendocrine carcinoma of the breast may prevail on a core biopsy samples increasing the probability of underdiagnosing this tumor preoperatively. Being aware of the existence of this disease allows for timely diagnosis and management. Optimal treatment requires simultaneous consideration of both the neuroendocrine and breast *in situ* tumor features.

## Background

Primary neuroendocrine carcinoma of the breast (NECB) was originally described in breast cancers with carcinoid-like growth patterns [1,2]. Subsequent reports have been used to define the common features of NECB by combining histologic findings with ultrastructural, molecular, and immunohistochemical data [3,4]. Because it can mimic some of the most common histologic subtypes of breast cancer, primary NECB is difficult to diagnose and therefore remains under-recognized. Herein we report the case of a patient initially diagnosed with invasive ductal carcinoma (IDC) that was postoperatively found to have a primary NECB. Additionally we provide a comprehensive review of the literature encompassing detailed information regarding epidemiology, histogenesis, clinical and histologic diagnosis criteria, classification, surgical and adjuvant treatment, as well as prognosis. We also provide recommendations for common clinical and histologic pitfalls.

## Case presentation

A 51-year-old Hispanic woman with no previous medical history presented with a self-detected lump in her right breast. Physical examination revealed a 2.0 cm firm mass in the outer quadrants of the right breast, which was adhered to the chest wall. The left breast and both axillae were normal. Mammography revealed a distinctive mass with microscopic calcifications and spiculation signs in the upper-outer quadrant of the right breast (BI-RADS 3). A core needle biopsy of the mass reported high-grade IDC. Staging workup, which included a chest CT and a liver ultrasound, was negative for metastatic disease.

The patient was staged with a locally advanced IIIB (T4aN0M0) breast cancer and underwent neoadjuvant therapy consisting of four cycles of doxorubicin and cyclophosphamide followed by 33 sessions of radiation therapy (total dose, 66 Gy to the site of the tumor and 50 Gy to the rest of the breast and axilla). During the course of treatment the tumor did not show any significant change in size; accordingly the patient underwent right modified radical mastectomy.

**Figure 1:**
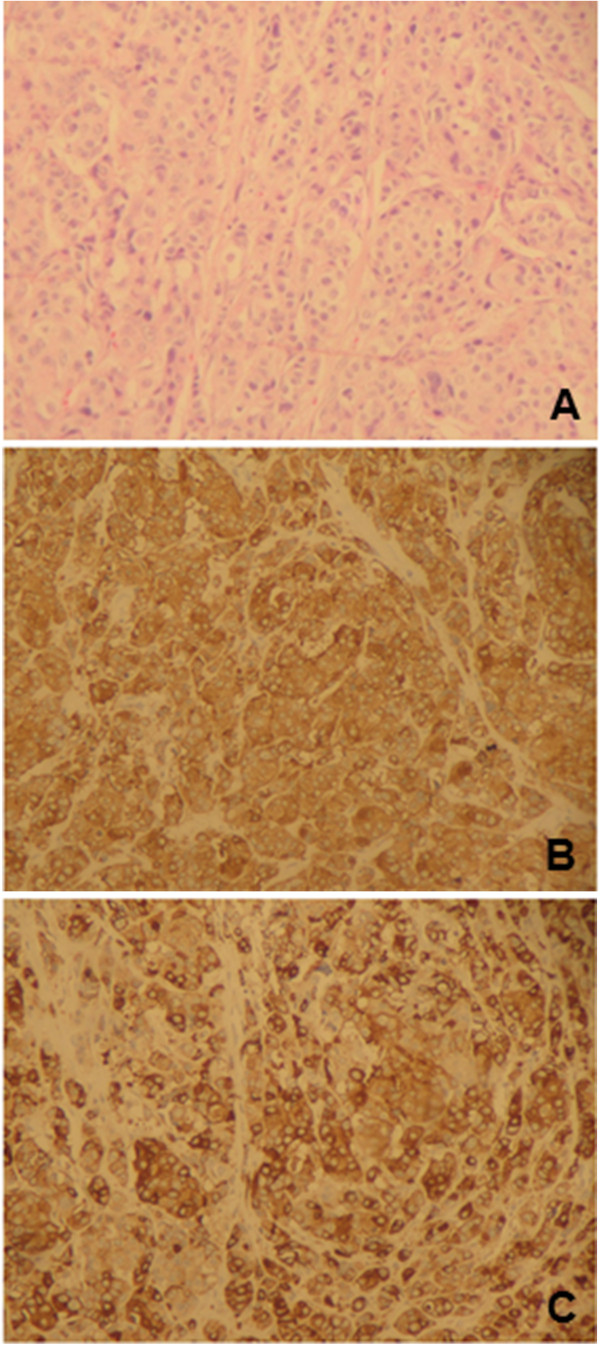
**Histopathological findings of the primary alveolar-type neuroendocrine carcinoma of the breast. **The tumor showed solid nests of cancer cells growing in alveolar-like patterns, which were separated by fibrovascular stroma and collagen and infiltrated the ducts and ductules (**A**). The neuroendocrine tumor component showed diffuse positive immunoreactivity to chromogranin A (**B**) and synaptophysin (**C**) (original magnification: A: 10× and B, C: 40×).

On gross examination, a 3.2×1.2 cm firm, grey mass with infiltrating margins was noted. Histopathologically the tumor was characterized by small, uniform cancer cells growing in nests and alveolar-like structures surrounded by delicate fibrovascular stroma and collagen that invaded ducts and ductules (Figure 1). Cancer cells were polygonal, round, and oval shape and had finely granular nuclear chromatin with uniform and vesicular nuclei and relatively eosinophilic cytoplasm. Due to these features immunohistochemistry (IHC) with neuroendocrine markers was performed. Cancer cells stained positive for both synaptophysin and chromogranin A (individual reactivity rate 100%) (Figure 1). A high-grade IDC component was also observed within the same tumor. Cancer cells were positive for estrogen receptors (ER) (reactivity rate 90%) and negative for progesterone receptors (PR) and HER-2 (HercepTest™ score 0). The Ki-67 proliferation index was >20%. Axillary lymph nodes did not harbor metastases. Based on these histological findings the tumor was classified as an alveolar-type invasive NECB with IDC.

Postoperatively, the patient was treated with three cycles each of cisplatin/etoposide followed by paclitaxel/carboplatinum. She was also started on hormone therapy (tamoxifen then switched to letrozole). Thirteen months after surgery the patient was alive, but she unfortunately developed pulmonary, bone, and hepatic metastases.

## Discussion

In the last 50 years histological and immunohistochemical studies have extended our knowledge about neuroendocrine tumors. Different diagnostic technologies allow us to distinguish pathological changes in endocrine cells. It is now possible to identify neuroendocrine features morphologically and biochemically within different histological types of invasive breast cancer. NECB was originally described in 1963 by Feyrter *et al.* when several cases of invasive breast cancer appeared to have a carcinoid growth pattern [1]. In 1977 Cubilla and Woodruff reported a case series of patients with breast tumors with the same features [2]. Later on in 1982 a modified silver stain (grimelius) and electron microscopy were both routinely used to identify neurosecretory granules and if present within a tumor the patient was diagnosed with ‘argyrophilic breast carcinoma’, a term coined by Azzopardi *et al.*[5]. Towards the end of the 1980s chromogranin and synaptophysin were found to be neuroendocrine differentiation markers and tumors that were once denominated ‘argylophilic breast carcinoma’ also tested positive for these markers [6]. It was only until 2002 when Sapino *et al.*[7] first suggested a specific definition for NECB, which was subsequently adopted by the World Health Organization (WHO) in 2003 as a means of endorsing it as a unique type of breast cancer [8]. In the WHO classification neuroendocrine tumors have been defined as those in which one or more neuroendocrine markers, such as neuro specific enolase, chromogranin A, and/or synaptophysin, are expressed in at least 50% of cancer cells [8]. In addition to this, diagnosing primary NECB also requires fulfilling two other criteria: (1) other primary sites must be ruled out and (2) the tumor must show histological evidence of a breast *in situ* component [9].

The prevalence of primary NECB was once reported to be as high as 12% to 19.5%, but this was based on early diagnostic criteria, various sources of tissue, and IHC neuroendocrine markers [10-13]. According to WHO diagnostic criteria the incidence is reported to actually range from 0.3% to 0.5% [14,15]. The peer-reviewed literature reveals that >80 patients have been reported (Table 1). A significant proportion (59.8%) of these cases was published after the WHO definition was established in 2003 allowing for consistency with respect to diagnostic criteria. The majority of cases have been described in women and so far only two cases in men have been reported [16,17]. The reported age of incidence ranges from 20 to 83 years with a higher incidence (60.2%) occurring in patients aged ≥50 years.

**Table 1:** **Summary of primary neuroendocrine carcinoma of the breast cases published in the indexed literature ( *n *=83)**^a^

						**Neuroendocrine IHC**					**Hormonal receptors**			**Treatment**				
**Author (year) [Ref]**	**Age (years)/Sex**	**Site**	**Size (cm)**^**b**^	**TNM**	**IS**	**GML**	**NSE**	**CgA/B**	**Syn**	**NSG**	**ER**	**PR**	**HER2/Neu**	**Neoadjuvant**	**Surgical**	**Adjuvant**	**Follow-up (months)**	**Outcome**
Wade (1983) [18]	52/F	R	10.0	T4N1M0	-	-				+	NS	NS	NS	NS	Mast/ALND	Ch	9	DOD
Jundt (1984) [16]	52/M	R	NS	TxN1M0	NS	-	+			+	NS	NS	NS	NS	NS	Ch/RT	14	DOD
Papotti (1992) [19]	64/F	R	2.0	T1N0M0	+	-	-	-	-		-	NS	NS	NS	Mast/ALND	NS	44	NED
	41/F	L	3.5	T2NxM0	+	-	+	-	-	+	-	NS	NS	NS	Mast/ALND	RT	15	DOD
	41/F	L	3.0	T2NxM0	+	+	+	+	+	+	-	NS	NS	NS	Mast/ALND	Ch	14	LR/DOD
	69/F	L	5.0	T3NxM0	+	+	+	-	+		+	NS	NS	NS	Mast/ALND	HT	9	DOD
Papotti (1993) [17]	83/M	R	1.5	T1N0M0	+	+	+	+			-	NS	NS	NS	Mast	NS	84	DOD
Francois (1995) [20]	68/F	R	4.5	T2N0M0	NS			+			-	-	NS	-	Mast/ALND	RT	21	D-NOS
Chua (1997) [21]	45/F	L	NS	T2N0Mx	NS	NS	NS	NS	NS	NS	NS	NS	NS	NS	BCS	NS	NS	NS
Fukunaga (1998) [22]	56/F	R	10.0	T3NxM0	+			+	+		-	-	NS	-	Mast/ALND	-	48	NED
Samli (2000) [23]	60/F	L	8.0	T4N1M0	NS			+	+		NS	NS	NS	Ch	Mast/ALND	Ch/RT	6	LR
Shin (2000) [24]	43/F	R	1.3	T1NxM0	NS		+				NS	NS	-	-	BCS	RT	30	NED
	44/F	L	2.0	T1NxM0	NS		+				NS	NS	-	-	BCS/ALND	Ch/RT	27	NED
	46/F	R	3.4	T2NxM0	NS		+				NS	NS	-	-	Mast/ALND	Ch	11	AWM
	50/F	R	2.2	T2NxM0	+		+				NS	NS	-	-	BCS/ALND	Ch/HT	35	NED
	51/F	R	1.5	T1NxM0	NS		+				NS	NS	-	-	BCS/ALND	RT	25	NED
	57/F	L	2.5	T2NxM0	NS		+				NS	NS	-	-	Mast/ALND	Ch	10	NED
	62/F	L	5.0	T3NxM0	+		+				NS	NS	-	Ch	Mast/ALND	Ch/HT	32	AWM
	64/F	L	1.8	T1NxM0	NS		+				NS	NS	-	-	BCS/ALND	Ch	10	NED
	70/F	L	4.0	T2NxM0	+		+				NS	NS	-	-	BCS/ALND	Ch/RT	3	NED
Yamasaki (2000) [25]	41/F	R	4.5	T2N0M0	+			+			-	NS	NS	-	BCS/Mast/ALND	Ch	16	NED
Salmo (2001) [26]	46/F	R	4.0	T2N0M0	+	+	NS	NS	NS	NS	-	+	NS	-	BCS	Ch/RT	9	NED
Zekioglu (2003) [27]	79/F	R	1.5	T1N0M0	+		+	+	-		+	+	NS	NS	Mast/ALND	NS	24	NED
	76/F	L	2.2	T2N0M0	+		+	+	+		+	+	NS	NS	BCS	NS	18	NED
	65/F	R	3.5	T2N0M0	+		+	-	+		+	+	NS	NS	Mast/ALND	NS	16	NED
	68/F	R	7.0	T3N1M0	+		+	+	+		-	-	NS	NS	Mast/ALND	NS	22	NED
	69/F	R	1.0	T1N0M0	+		+	+	+		+	+	NS	NS	Mast/ALND	NS	12	NED
	72/F	R	1.5	T1N0M0	+		+	-	+		+	+	NS	NS	BCS	NS	13	NED
	60/F	R	1.0	T1N0M0	+		+	+	+		+	+	NS	NS	BCS	NS	10	NED
	43/F	L	0.8	T1N0M0	+		+	-	+		+	+	NS	NS	BCS	NS	48	NED
	60/F	L	2.5	T2N0M0	+		+	-	+		+	+	NS	NS	Mast/ALND	NS	54	NED
	43/F	L	5.0	T2N0M0	+		+	-	+		+	+	NS	NS	BCS	NS	NS	NED
	72/F	L	2.7	T2N0M0	+		+	-	+		+	+	NS	NS	BCS	NS	NS	NED
	72/F	L	2.7	T2N0M0	+		+	-	+		+	+	NS	NS	Mast/ALND	NS	NS	NED
Bergman (2004) [28]	61/F	L	2.5	T2NxM0	+		+	-	-		-	-	-	-	Mast/ALND	NS	NS	NS
Bigotti (2004) [29]	56/F	L	18.0	T3N1M0	+		+	-	+		-	-	-	Ch	Mast	Ch	14	D-N0S
Jochems (2004) [30]	71/F	NS	3.0	T2NxM0	NS		+	+	+		+	+	-	-	Mast/ALND	HT	12	NED
Sridhar (2004) [31]	58/F	R	2.0	T1NxM0	NS			-			-	-	NS	-	BCS/ALND	Ch/RT	18	NED
Yamamoto (2004) [32]	53/F	NS	6.5	T3N2M0	NS		+				NS	NS	NS	NS	NS	NS	34	NED
	75/F	NS	2.0	T2N1M0	NS		+				NS	NS	NS	NS	NS	NS	43	NED
Berruti (2004) [33]	59/F	NS	NS	T2N0M0	+		+	+	+		+	-	NS	-	Mast	HT	NS	AWM
Adegbola (2005) [34]	46/F	R	1.0	T1N0M0	NS			+	+		-	-	-	-	BCS	Ch/RT	48	NED
	60/F	R	1.7	T1NxM0	+			+	+		-	-	-	-	BCS	Ch/RT	20	D-NOS
	61/F	L	1.7	T1NxM0	NS			+			-	-	-	-	BCS	Ch/RT	6	AWM
Valdes (2006) [35]	41/F	R/MF	1.5	T1NxM0	+				+		-	-	NS	-	Mast/SLNB/Re	Ch	NS	NS
Fujimoto (2007) [36]	40/F	L/MF	2.0	T1NxM0	+			+	+		+	+	+	-	Mast/SLNB	HT	36	NED
Kim (2008) [37]	27/F	L	3.2	T2NxM0	NS		+	+	+		NS	NS	NS	-	BCS/ALND	Ch/RT	18	NED
Kinoshita (2008) [9]	31/F	L	6.0	T3NxM0	+		+	+			-	-	-	Ch	Mast/ALND	Ch	9	D-NOS
Stita (2009) [38]	64/F	L	3.0	T2NxM0	+		+	+			+	+	NS	-	Mast/ALND	Ch	8	NED
Yamaguchi (2009) [39]	51/F	R	3.0	T2NxM0	NS			+	+		-	-	-	-	Mast/ALND	Ch	12	AWM
Christie (2010) [40]	61/F	L	3.0	T2NxM0	+			+	+		-	-	-	-	BCS/ALND	Ch	3	D-NOS
Latif (2010) [41]	53 /F	R	6.0	T3N0M0	NS			+	+		-	-	-	Ch	BCS/SLNB	RT	NS	NS
Honami (2011) [42]	54/F	B	L: 1.0	T1N0M0	L: +			+	+		+	+	-	-	BCS	RT	18	NED
			R: 1.5		R: +													
Kawanishi (2011) [43]	67/F	R	0.8	T1NxM0	+			+	+		+	+	-	-	BCS/ SLNB	HT	12	NED
Nozoe (2011) [44]	57/F	R	3.0	T2NxM0	NS		+		+		+	+	-	-	Mast/ALND	Ch	NS	NS
Zhang (2011) [45]	29/F	B	L: 8.5	T1N2M0	L: +		+	+	+		+	+	-	-	BCS	Ch	NS	NS
			R: 2.0		R: +													
Su (2012) [46]	75/F	L	4.0	T2N0M0	NS			+	+		+	+	-	-	Mast/ALND	HT	20	NED
Miura (2012) [47]	72/F	R/MC	1.5	T1N0M0	Both +			+	+		+	+	-	NS	Mast/SLNB	NS	NS	NS
Kawasaki (2012) [48]	41/F	L	0.0	TisN0M0	+			+	+		+	+	NS	NS	BCS	NS	101	NED
	45/F	R	0.0	TisN0M0	+			+	+		+	+	NS	NS	BCS	NS	80	NED
	41/F	L	0.0	TisN0M0	+			+	+		+	+	NS	NS	BCS	NS	90	NED
	74/F	R	0.0	TisN0M0	+			+	+		+	+	NS	NS	BCS	NS	91	NED
	28/F	R	0.0	TisN0M0	+			+	+		+	+	NS	NS	Mast	NS	77	NED
	30/F	L	0.0	TisN0M0	+			+	+		+	+	NS	NS	BCS	NS	86	NED
	58/F	R	0.0	TisN0M0	+			+	+		+	+	NS	NS	BCS	NS	96	NED
	36/F	L	0.0	TisN0M0	+			+	+		+	+	NS	NS	BCS	NS	64	NED
	38/F	R	0.0	TisN0M0	+			+	+		+	+	NS	NS	Mast	NS	69	NED
	60/F	L	0.1	T1N0M0	+			+	+		+	+	NS	NS	BCS	NS	84	NED
	42/F	L	0.1	T1N0M0	+			+	+		+	+	NS	NS	Mast	NS	73	NED
	43/F	R	0.1	T1N0M0	+			+	+		+	+	NS	NS	BCS	NS	80	NED
	35/F	R	0.1	T1N0M0	+			+	+		+	+	NS	NS	BCS	NS	100	NED
	70/F	L	0.1	T1N0M0	+			+	+		+	+	NS	NS	Mast	NS	93	NED
	72/F	R	0.1	T1N0M0	+			+	+		+	+	NS	NS	Mast	NS	66	NED
	62/F	L	0.1	T1N0M0	+			+	+		+	+	NS	NS	BCS	NS	88	NED
	38/F	R	0.2	T1N0M0	+			+	+		+	+	NS	NS	BCS	NS	85	NED
	73/F	R	0.3	T1N0M0	+			+	+		+	+	NS	NS	Mast	NS	71	NED
	43/F	L	0.4	T1N1M0	+			+	+		+	+	NS	NS	BCS	NS	86	NED
	42/F	L	0.5	T1N0M0	+			+	+		+	+	NS	NS	BCS	NS	96	NED
	39/F	R	0.5	T1N0M0	+			+	+		+	+	NS	NS	Mast	NS	74	NED
	33/F	R	0.7	T1N0M0	+			+	+		+	+	NS	NS	Mast	NS	92	NED
	36/F	L	1.5	T1N0M0	+			+	+		+	+	NS	NS	BCS	NS	99	NED
	68/F	R	2.5	T2N0M0	+			+	+		+	+	NS	NS	Mast	NS	68	NED
(2012) [Present case]	51/F	R	2.0	T4aN0M0	+			+	+		+	-	-	Ch/RT	Mast/ALND	Ch/HT	13	AWM

^a^Studies included in this table are those available in PubMed as of May 31, 2012. Papers that were excluded from this summary are those in which information was not provided explicitly in the description of the case report or because the results were grouped into cohorts [12,49,57].

^b^Obtained either from clinical history or the final pathology report; for multifocal or multicentric disease the largest tumor was registered.

ALND, axillary lymph node dissection; AWM, alive with metastatic disease; B, bilateral; BCS, breast conserving surgery; CgA/B, chromogranin A and/or B; Ch, chemotherapy; D-NOS, dead, but cause not otherwise specified; DOD, dead of other disease; ER, estrogen receptors; F, female; GML, Grimelius staining; HT, hormone therapy; IHC, immunohistochemistry; IS, breast *in situ* component; LR, local recurrence; M, male; Mast, mastectomy; MC, multicentric; MF, multifocal; NED; no evidence of disease; NS, information not specified; NSE, neuron-specific enolase; NSG, neurosecretory granules; PR, progesterone receptors; R, right; Re, reconstruction; RT, radiation therapy; SLNB, sentinel lymph node biopsy; Syn, synaptophysin.

Patients with primary NECB do not have any distinctive clinical presentation (Table 2). Indeed on clinical examination the findings are similar to those of any other type of invasive breast cancer. Nodule(s) size ranges from Tis to 18.0 cm and are not generally associated with axillary lymphadenopathy. The majority of tumors are <2.0 cm and patients are staged with early breast cancer. The radiological features may include a highly-dense mass with a spiculated or microlobulated margin on mamography and/or a homogenously hypoechoic massive lesion with normal sound transmission on ultrasonography [14]. It is also possible for it to be mistaken for benign disease such as fibroadenomas or cysts because the tumor may have clear-cut, circumscribed borders [14]. Distinguishing primary from metastatic NECB is not achieved with breast imaging [49].

**Table 2:** Representative clinical and histopathological features of primary neuroendocrine carcinoma of the breast

**Characteristic**	**Features**
**Epidemiologic**	
Age of diagnosis (years)	>50
Sex	Female>Male
**Physical examination**	
Clinical presentation	Single palpable, well-circumscribed nodule (x̅: 2.5cm) or nipple discharge.
Nodal status	Non-palpable axillary lymph nodes
Carcinoid symptoms	Absent
**Histopathology**	
Tumor components	Co-existing neuroendocrine and ductal cancer cell populations possibly from divergent differentiation of cancer stem cells (lobular or other types of breast cancer are rare).
Multifocality or multicentricity	Rare
Growth pattern	Solid carcinoid-like (most common), large cell-type, and small/oat cell-type
Cell type	Homogenous group of plasmacitoid, signet ring, clear cell, or small/oat cells
Histopathological features	Nuclear palisading; pseudorosette formation; loss of cell cohesion; intra- and/or extra-cellular mucin content; and abundant eosinophilic cytoplasm and nuclei with stippled (‘salt and pepper’) chromatin.
Diagnostic markers	Most sensitive and specific: chromogranin A or B and synaptophysin.
	Least specific: neurospecific enolase, CD56, neurofilament triplprotein, and bombin or leu.
Hormonal receptors	Estrogen/progesterone receptor positive
	HER2 negative
Molecular subgroup	Luminal A (basal-type has been documented)
**Staging**,	TisN0M0: 9 (10.9)
***n*** (%) (***N***=82)^a^	T1NxM0: 35 (42.7)
	T2NxM0: 27 (32.9)
	T3NxM0: 8 (9.8)
	T4NxM0: 3 (3.7)

^a ^Based on case reports that specified size of the lesion; includes present case.

Before any further action is taken the NECB must be classified as ‘primary’ or ‘metastatic’. Physicians should primarily focus on ruling out breast metastasis from small cell carcinoma of the lung, the gastrointestinal tract, pancreas, and the cervix. Metastatic neuroendocrine carcinoma from other organs cannot be distinguished from primary NECB because histologically they are similar [50]. In this case workup with imaging is fundamental. Differential diagnoses should also include Merkel cell carcinoma, lymphoma, carcinoid tumor, and melanoma [41]. Combining clinical data, radiologic findings, and immunohistochemical stains specific to each tumor provides sufficient evidence to rule out metastatic sources of NECB and other primary tumors [11].

Tumors with neuroendocrine immunoreactivity below the 50% threshold should be considered to have focal neuroendocrine differentiation. Generally these are IDC-not-otherwise specified (IDC-NOS) although lobular or medullary carcinomas can also present this feature. Breast cancers with focal neuroendocrine differentiation typically have 12% to 18% of IHC-positive neuroendocrine cells [12,13,51]. These tumors tend to resemble common histologic types of breast cancer rather than NECB in terms of age of presentation, size, histologic grade, and lymph nodal status [10].

The histogenesis of NECB has not been fully clarified. An initial theory suggested that NECB cancer cells derived from argyrophilic cells of neural crest origin that migrated to the mammary ducts [2]. Another theory supports *in situ* development from neuroendocrine cells naturally found in the breast, but these cells have not been consistently found by other authors [4,52]. A novel hypothesis suggests that NECB results from an early divergent differentiation event in breast carcinogenesis in which neoplastic stem cells differentiate into both epithelial and endocrine lines [10,53]. Molecular analysis studies support this theory because studies have shown that the neuroendocrine component is clonally related to the intraductal component [54] and that the whole tumor itself is of the luminal subtype [55].

Histologically describing the breast *in situ* component is important because this determines which adjuvant treatment regimens are chosen. Simultaneous presence of an intra-ductal component and the absence of other primary sites establish the breast as the primary organ of origin [11,54]. Unlike the histogenesis of other common types of neuroendocrine carcinoma where there is evidence of benign neuroendocrine tumors these precursor lesions are extremely rare in the breast. Of the case reports published so far, only one has described a tumor with co-existing NECB and neuroendocrine cell hyperplasia [47]. Kawasaki *et al.* has also described that neuroendocrine ductal carcinoma *in situ* (DCIS) could be considered a pre-invasive stage of NECB [56]. Neuroendocrine DCIS are frequently underdiagnosed preoperatively because it resembles ductal hyperplasia and intraductal papilloma and sampling is quite difficult [56].

Primary NECB is not associated with any definitive gross pathological characteristics. The breast *in situ* component of primary NECB is usually found as an intraductal lesion co-existing with the neuroendocrine carcinoma component [49].

The breast *in situ* component consistently has histopathological features that include large or dilated ducts with the luminal spaces completely filled; distinctive cells with ovoid, polygonal, and spindle shapes; and a low- or moderate- grade of nuclear atypia [49,57]. Additionally this specific component creates four pitfalls during diagnosis: (1) the invasive component of primary NECB can mimic DCIS [57]; (2) non-specific glandular patterns within the tumor can lead to a diagnosis of IDC-NOS [51,57]; (3) cases of invasive lobular carcinoma or carcinoma with lobular features may not be recognized as having neuroendocrine differentiation [57]; and (4) the intraductal component of primary NECB may be mistaken for atypical intraductal hyperplasia or atypical papilloma [49,57]. In our case we initially overlooked this tumor because the IDC component prevailed on the core biopsy sample and final surgical pathology was necessary to obtain a definitive diagnosis. Over two-thirds of the cases in the literature report initial misdiagnosis later rectified after surgery [57].

Histologically the neuroendocrine component resembles lung and gastrointestinal neuroendocrine tumors. It is characterized by cellular monotony, nuclear palisading, pseudorosette formation, loss of cell cohesion, and abundant eosinophilic cytoplasm and nuclei with stippled (‘salt and pepper’) chromatin [49,57-59]. Nevertheless these features *per se* are not sensitive enough to rule in a diagnosis because they are inconsistently present [57]. Pathologists mainly rely on using a panel of the most sensitive and specific IHC neuroendocrine markers (chromogranin A or B and synaptophysin) [51]. Other less specific markers, such as neurospecific enolase [12], CD56 [15], neurofilament triplprotein, and bombin or leu [12], should be avoided. Negative immunoreactivity can be common even in in tumors with a distinctive morphologic growth pattern strongly suggestive of NECB [51,60]. In such cases, further analyses with techniques such as *in situ* hybridization [61] and second opinion are valid options.

Primary NECB comprises a heterogeneous group of histologic subtypes that differ from one another depending on the prevalent growth pattern, cell type, level of invasiveness, and prognosis [62]. Several classifications have been proposed [3,63]. The WHO eventually proposed three histologic categories (solid carcinoid-like, large cell-type, and small/oat cell-type) [8], which they derived from the fact that NECB resembles high-grade small and large cell neuroendocrine pulmonary carcinoma. On another note the concomitant presence of neuroendocrine features and mucin differentiation within the same tumor and even in the same cell (amphicrine cell) is almost exclusive to NECB [15,51]. This characteristic led several authors to establish mucin-producing subtypes [17,51,64]. Although the WHO does recognize the fact that mucinous differentiation occurs in 26% of patients with NECB no changes have been made to their classification. Recently, primary NECB has been classified in terms of molecular taxonomy. Gene expression profiling analysis has shown that this group of tumor is of the luminal type [55]. Basal-type characteristics have only been reported in one patient [65].

Patient outcome is not affected by the size of the neuroendocrine component [10]; instead three histological parameters (histological grade, mucin production, and apocrine differentiation) have more significant impact. The most important histological factor is the histological grade [66], which is to some extent related to the histologic subtype. For example, solid neuroendocrine carcinoma and atypical carcinoids, described as well-differentiated tumors, have a better prognosis [38] than small cell and large cell NECB, which are poorly differentiated and have an unfavorable prognosis [67]. Our case highlights the impact histological grade has on a patient’s outcome as we believe this may explain why the patient progressed to metastasis despite receiving optimal treatment. Mucin production is highly relevant because solid papillary carcinomas and mucinous carcinomas produce significantly longer survival times than other subtypes of NECB with no mucin content [67,68]. Similarly the presence of apocrine differentiation has correlated with a better prognosis [69].

Specific recommendations regarding surgical management do not exist. Patients should be treated similarly to IDC whose choice of surgical procedure depends on the tumor’s location and clinical stage [12,49,70]. Differentiating ‘primary’ from ‘metastatic’ NECB is crucial because the latter does not justify submitting a patient to mastectomy and axillary node dissection [49]. Of the patients reported in the literature 48.3% have undergone mastectomy and 40% axillary lymph node dissection. Confirming negative surgical margins can be difficult, especially from intraoperative frozen sections, because NECB may have pagetoid involvement or a background of neuroendocrine cell hyperplasia can produce artifacts [47,56]. To date there is limited information in the literature regarding safety of oncoplastic breast conservation and immediate breast reconstruction. Thus far only one study has reported the case of a patient that underwent mastectomy followed by deep inferior epigastric perforator flap reconstruction, however the authors failed to describe margin status and disease-free survival [35]. Given the difficulty in assessing tumor margin status for this type of tumor, oncoplastic breast conservation and immediate breast reconstruction may not be beneficial to some patients with primary NECB.

The real challenge with primary NECB lies in choosing the ideal type of cytotoxic therapy. Currently there is no information that indicates what the most efficacious regimen is, but the general consensus is to treat it with chemotherapy regimens for common histologic types of breast cancer [11,15,62,64] and pulmonary small cell carcinoma neuroendocrine carcinoma [11,41,71]. Of the cases that give detailed information on treatment (*n*=39), 28 received neo- and/or adjuvant chemotherapy. Some examples of the chemotherapy regimens reported in the literature include fluorouracil/epirubicin/cyclophosphamide followed by docetaxel; etoposimide and carboplatin or cisplatin; cisplatin/irinotecan; adriamycin and cytoxan or cisplatin; paclitaxel alone; and cyclophosphamide/methotrexate/fluorouracil. Our patient was treated with cisplatin/etoposimide followed by paclitaxel/carboplatinum; the former combination is the most widely used in primary NECB. Radiation therapy appears to be used to a lesser extent than chemotherapy; only 18 patients have received it alone or in combination with chemotherapy.

Because breast biomarkers have only recently become a standard in pathology reports many of the earlier case reports lack this information. As seen in Table 1 primary NECB tends to heterogeneously express ER, PR, and HER-2; this may explain why the overall outcome of these patients varies so much between different cohorts. Of the case reports with complete hormone receptor information available (*n*=18) 9 were triple negative, 7 were ER/PR positive and HER-2 negative, and the remaining 2 had other combinations. In this current case report the patient’s tumor was ER positive and PR and HER-2 negative and hormone therapy was accordingly prescribed. The current recommendation is for patients with primary NECB to receive hormone therapy based on their hormonal receptor status [49].

At the time of publication of each case report (*n*=83) 58 patients (69.9%) had no evidence of disease, 8 (9.6%) were alive with local recurrence or metastasis, 5 (6.0%) died of other disease, and 12 (14.5%) died of a cause not specified. Only four cases (4.8%), including ours, of locally-advanced primary NECB have been described, but ours was the only to progress to metastatic disease. In general primary NECB, particularly the small cell carcinoma subtype, is as aggressive as pulmonary neuroendocrine carcinoma [11,24,29]. Both these tumors are characterized by their resistance to multi-modal cytotoxic therapies [71]. Our patient is a clear example of this because although she was treated accordingly limited benefit was observed as she quickly developed widespread metastases.

The few studies that give insight into outcome have mixed results. A study by van Krimpen *et al.* suggested that the prognosis does not differ from that of the more common types of breast cancer [13]. This is in accordance with Miremadi *et al.*’s study that reported that cases of primary NECB have the same mean age, size, histologic grade, nodal status, and prognosis as their non-primary NECB counterparts [10]. It has also been suggested that patients with primary NECB of non-small cell type may even have a better prognosis than patients with IDC or invasive lobular carcinoma [15,72]. Alternatively other studies have described a worse outcome overall [62,73].

Several unsolved issues are frequently discussed in the literature. First of all case series have only analyzed their patients as a homogenous population without taking into consideration the different histologic subtypes [11,51]. Second, standard prognostic parameters, specifically histologic grade, are not consistently taken into account when comparing primary NECB cases with non-NECB [11,51]. In addition to this randomized clinical trials comparing the different treatment regimens and their outcomes have not been carried out. All these unsolved matters are obviously related to the rarity of this tumor. Interestingly enough many of the studies included in this review did not provide essential clinical, histologic, diagnostic, and therapeutic information in their case report, which makes comparing them difficult. In this respect if future cases of primary NECB could be prospectively and collectively registered in a single international database encompassing standard epidemiological information and stratifying patients according to histological and molecular subtype then physicians would have a valuable tool to truly understand this tumor.

## Conclusion

Primary NECB has been sporadically reported in the literature since 1963, but formal diagnostic criteria have only been available since 2003. Although primary NECB may have morphological characteristics that resemble classic neuroendocrine tumors the histopathological diagnosis can only be made with neuroendocrine markers. Given that a breast *in situ* component may prevail on a core biopsy samples primary NECB may be easily overlooked preoperatively. Being aware of the existence of this disease may allow for timely diagnosis. Misdiagnosing primary NECB is detrimental because patients may not receive the optimal adjuvant treatment they need. Treating a patient with primary NECB requires simultaneous consideration of both the neuroendocrine and breast *in situ* tumor components. At the moment it is difficult to fully understand this rare tumor because issues such as histogenesis, optimal adjuvant therapy, and prognosis are still unknown. The limited number of patients in the literature have presented in different clinical stages and received different treatment combinations thus the data summarized in this review should be interpreted cautiously.

## Consent

Written informed consent was obtained from the patient for publication of this case report and any accompanying images. A copy of the written consent is available for review by the Editor-in-Chief of this journal.

## Competing interests

The authors declare that they have no competing interests.

## Authors’ contributions

FAA obtained medical history, searched and reviewed the literature, drafted the manuscript, and edited the final version; JLR obtained patient follow-up information, carried out the histopathological studies, and edited the final version; EM carried out the histopathological studies, provided diagnostic consultation, and edited the final version; JOS obtained medical history, provided diagnostic consultation, managed the patient, and edited the final version; MT provided diagnostic consultation, managed the patient, and edited the final version; and LT obtained medical history, provided diagnostic consultation, managed the patient, searched literature, and edited the final version. All authors read and approved the final manuscript.
